# Accuracy Assessment of iPhone LiDAR for Mapping Streambeds and Small Water Structures in Forested Terrain

**DOI:** 10.3390/s25196141

**Published:** 2025-10-04

**Authors:** Dominika Krausková, Tomáš Mikita, Petr Hrůza, Barbora Kudrnová

**Affiliations:** 1 Department of Forest Management and Applied Geoinformatics, Faculty of Forestry and Wood Technology, Mendel University in Brno, Zemědělská 3, 613 00 Brno, Czech Republic; dominika.krauskova@mendelu.cz (D.K.); xkudrno1@mendelu.cz (B.K.); 2 Department of Landscape Management, Faculty of Forestry and Wood Technology, Mendel University in Brno, Zemědělská 3, 613 00 Brno, Czech Republic; petr.hruza@mendelu.cz

**Keywords:** LiDAR mapping systems, water structures, mobile laser scanning, point cloud, iPhone LiDAR

## Abstract

Accurate mapping of small water structures and streambeds is essential for hydrological modeling, erosion control, and landscape management. While traditional geodetic methods such as GNSS and total stations provide high precision, they are time-consuming and require specialized equipment. Recent advances in mobile technology, particularly smartphones equipped with LiDAR sensors, offer a potential alternative for rapid and cost-effective field data collection. This study assesses the accuracy of the iPhone 14 Pro’s built-in LiDAR sensor for mapping streambeds and retention structures in challenging terrain. The test site was the Dílský stream in the Oslavany cadastral area, characterized by steep slopes, rocky surfaces, and dense vegetation. The stream channel and water structures were first surveyed using GNSS and a total station and subsequently re-measured with the iPhone. Several scanning workflows were tested to evaluate field applicability. Results show that the iPhone LiDAR sensor can capture landscape features with useful accuracy when supported by reference points spaced every 20 m, achieving a vertical RMSE of 0.16 m. Retention structures were mapped with an average positional error of 7%, with deviations of up to 0.20 m in complex or vegetated areas. The findings highlight the potential of smartphone LiDAR for rapid, small-scale mapping, while acknowledging its limitations in rugged environments.

## 1. Introduction

With the increasing impacts of climate change, including more frequent torrential rains, the demand for water retention in the landscape is also rising. In addition to the construction of large water reservoirs (dams), nature-friendly modifications of small streambeds, the construction of structures, the adjustment of the longitudinal profile of the stream, or revitalization and, conversely, the return to the original riverbed are increasingly being promoted. In addition to constructing new structures on the watercourse, regular maintenance and monitoring of changes in the structures and the riverbed are also very important. For planning such modifications, capturing the current state is crucial for subsequent project design [1,2,3]. Tacheometric surveying using a total station is one of the most accurate methods [4,5]; however, it is time-consuming and cannot comprehensively capture the terrain’s profile. Additionally, the purchase of equipment incurs extra costs. On the other hand, GNSS devices enable faster surveying, but only in open terrain. In the case of small streambeds in forested areas, GNSS is often completely hindered by signal obstruction from tree canopies [6,7].

Very rapid and detailed mapping is possible using Terrestrial Laser Scanning (TLS), which captures the spatial coordinates with sub-millimeter precision [8]. This method efficiently captures 3D data over surveyed areas and features, with tens to hundreds of thousands of measurements per second. Its non-contact nature prevents damage or interference [9] and remains stable in challenging conditions, such as intense lighting or complex backgrounds [10]. Therefore, TLS has numerous applications across various fields, including archeology [11], geology [12], engineering [13], forestry [14], and environmental sciences [15], among others. It is also commonly used in fluvial applications to study erosion, streambank retreat, and the evolution of gully and channel morphology [16]. The main disadvantage of TLS for extensive area mapping is the need to scan from multiple positions. Increasing the number of scans or adjusting scanner settings can lead to data redundancies, making storage and management challenging. Larger datasets require more time and labor to collect, increasing costs [17].

The disadvantages of TLS for mapping surveyed areas and features are overcome by using Mobile Laser Scanning (MLS) or its variant, Personal Laser Scanning (PLS). While MLS is commonly used for outdoor point cloud acquisition, in the field, it faces challenges like GNSS limitations, making it less practical for outdoor use. Simultaneous Location and Mapping (SLAM) is being explored as an alternative to GNSS for outdoor use. Recent studies have shown that PLS systems can achieve comparable accuracy to TLS in many practical applications [18,19,20].

In addition to ground-based methods, aerial approaches using UAVs rely on RGB cameras with Structure from Motion (SfM) processing [21]. Still, dense vegetation often prevents reliable ground mapping, even when flown at low altitudes or outside the leaf-on period [22,23]. Drone-based LiDAR addresses this by penetrating canopies to capture bare-earth points, offering improved detail for streambeds and small structures such as retention structures [24].

The rapid development of LiDAR (Light Detection and Ranging) technology has shifted from expensive terrestrial laser scanners (TLS) to more affordable, portable sensors integrated into smartphones [25,26,27,28]. This evolution has made LiDAR technology more accessible, enabling it to map streambeds and small water structures—tasks traditionally reliant on complex geodetic methods [29]. Recent studies confirm that mobile LiDAR can generate high-resolution topographical models under specific conditions. For example, researchers [30,31] evaluated LiDAR for environmental monitoring, demonstrating its ability to map small-scale geomorphic features. The first applications of iPad LiDAR in forestry were presented in [32], showing its potential for tree inventory and terrain mapping. However, its accuracy diminishes over greater distances and under challenging conditions, such as those involving landscape morphology or dense vegetation. This limitation necessitates the development of refined scanning strategies tailored to the environment.

Advances in LiDAR point cloud processing algorithms and software, such as those integrated into mobile apps like 3D Scanner [33] and SiteScape [34], enable seamless alignment and fusion of multiple scans. Additionally, the 3D Scanner is free to use, making it particularly accessible for many users. This has implications for mapping features like streambeds, where detailed cross-sectional and areal scans are necessary to capture subtle geomorphological changes [35,36,37,38]. When integrated with traditional geodetic tools, mobile LiDAR can be a complementary method for verifying specific features or rapidly surveying areas that are otherwise difficult to access. Recent developments also include the use of Apple Vision Pro Goggles for environmental scanning [39]. These advantages suggest that mobile LiDAR is particularly well-suited for forested stream corridors, where capturing both hydrological features (streambeds, banks, and retention structures) and forestry attributes (tree density, root exposure, and road condition) is necessary.

Environmental conditions remain a critical factor in LiDAR performance. Dense vegetation, varying light conditions, and reflective water surfaces introduce noise, reducing data quality [40]. Structured scanning methodologies are necessary to address these challenges. Optimizing scanning angles, incorporating reference points, and conducting multiple scanning passes can significantly enhance data reliability, particularly in forested stream corridors. The deployment of mobile LiDAR in hydrological studies is gaining traction as more researchers recognize its potential for monitoring water flow, sediment transport, and erosion processes in small catchments [41]. Authors in a study [37] evaluated iPhone LiDAR in gravel-bed streams, finding it capable of capturing channel morphology and significant roughness elements with millimeter accuracy, although finer roughness elements were simplified. By enabling the quick generation of 3D models of water structures, this technology supports more dynamic and responsive water resource management strategies. As mobile LiDAR continues to evolve, its integration with other technologies, such as photogrammetry, UAVs (Unmanned Aerial Vehicles), and GNSS, can further enhance its applicability, creating a comprehensive suite of tools for environmental monitoring and management [36,42,43,44]. In forestry, iPhone LiDAR scanning apps have been shown to capture forest road profiles with centimeter-level accuracy, providing a cost-effective tool for monitoring and maintenance, though not as precise as TLS [38]. Such results highlight both the promise and limitations of mobile LiDAR in representing micro-topography, which is critical for flow resistance, sediment transport, and terrain condition monitoring. The innovative contribution of this work lies in evaluating the iPhone 14 Pro LiDAR for mapping streambeds and small water retention structures under complex conditions of dense vegetation and steep terrain, developing and comparing several tailored scanning workflows (area, cross-sectional, sectional, and structural scanning), and validating the results against precise geodetic measurements with systematically placed reference points along the stream. This combination of application domain, workflow design, and rigorous benchmarking provides new insights into the feasibility and limitations of smartphone LiDAR for watercourse monitoring. Despite limitations in long-range accuracy and environmental constraints, the portability, accessibility, and cost-effectiveness of mobile LiDAR make it a valuable addition to the geospatial toolkit.

The objective of this study is to evaluate the feasibility and accuracy of using iPhone 14 Pro LiDAR for mapping streambeds and small water structures in forested and rugged terrain. The research question is: Can mobile LiDAR integrated into smartphones provide comparable accuracy to traditional geodetic methods under varying environmental conditions? We hypothesize that mobile LiDAR, when supported by geodetic reference points, can achieve sufficient positional and vertical accuracy for practical applications in environmental monitoring. We assume that the iPhone 14 Pro, equipped with a LiDAR sensor, can serve as a viable alternative to GNSS receivers and total stations, delivering comparable positional and vertical accuracy while offering advantages in accessibility and time efficiency. This includes comparing mobile LiDAR data to geodetic measurements under various environmental conditions and use cases, such as mapping stream beds, small structures (e.g., retention structures), and adjacent infrastructure like forest roads. Additionally, it explores optimal data collection workflows, identifying configurations that strike a balance between accuracy and efficiency. By addressing these goals, the research aims to gain a clear understanding of the strengths and limitations of mobile LiDAR, thereby positioning it as a valuable tool for geospatial data collection while identifying areas for further technological improvement.

Unlike previous studies that focus on controlled environments or single scanning workflows, this study introduces a comparative evaluation of four distinct scanning procedures using a consumer-grade smartphone LiDAR in a forested stream corridor. The innovative aspect lies in integration with geodetic reference points under real-world conditions, enabling a detailed analysis of positional and vertical accuracy across varying terrain complexities. This structured approach provides practical insights into the feasibility of smartphone-based mapping for hydrological and environmental applications.

Additionally, the paper proposes a structured scanning workflow that includes multiple tailored scan modes. Hence, the work advances cost-effective and practical methodologies to leverage mobile LiDAR for environmental monitoring and stream management.

## 2. Materials and Methods

### 2.1. Study Area

**Figure 1:**
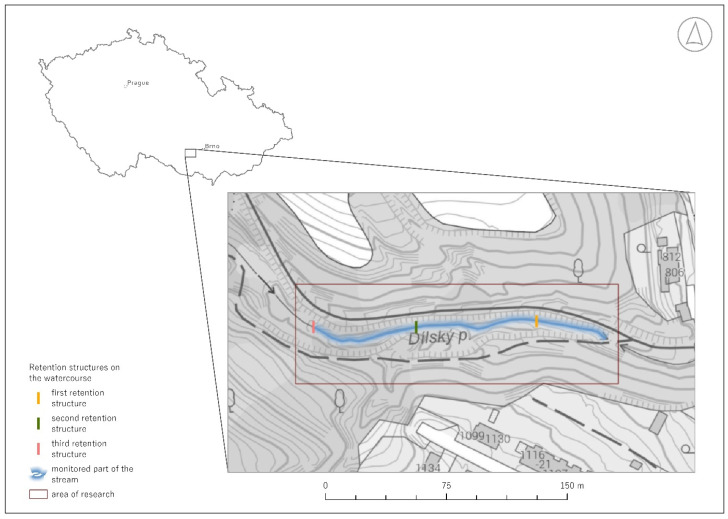
Research area of the monitored streambed with individual retention structures.

The study was conducted in an area located in the western part of the South Moravian region (Czech Republic), in the cadastral area of Oslavany (Figure 1). The monitored streambed is situated in the northwest part of the cadastral area, totaling 1.7 km. However, due to the complexity of the terrain, only a part of the stream was addressed, together with three retention structures. The section under consideration begins with a culvert that crosses a forest path, and the end of the measured stream is located at the last stone retention structure, where a large amount of water is retained. The total length of the measured section is 190 m—the mapped area around the streambed covered just under 2000 square meters.

### 2.2. Data Collection and Technologies Used

**Figure 2:**
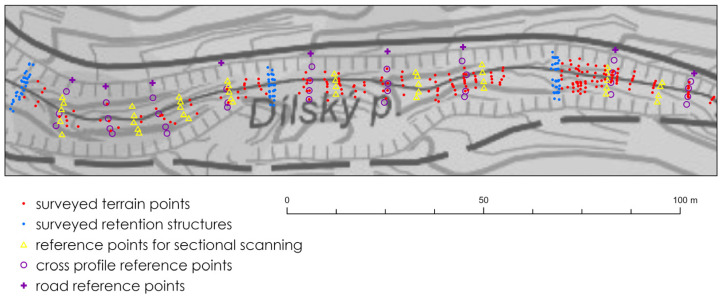
Measured terrain and reference points on the streambed along the forest road.

This study demonstrates the potential of smartphone LiDAR when applied not only to terrain mapping but also directly to streambed and hydraulic structure surveying, where challenges such as water reflection, complex streambed relief, and limited accessibility have so far constrained the use of low-cost LiDAR technologies. Data collection was conducted during a non-vegetative period, as dense vegetation surrounding the streambed can hinder the acquisition of undistorted scans and significantly impede movement within the streambed. Traditional geodetic surveying was conducted using a Trimble M3 total station (Trimble Inc., Sunnyvale, CA, USA) and a Trimble R12i GNSS receiver (Trimble Inc., Sunnyvale, CA, USA). GNSS measurements were conducted exclusively using fixed solutions, with an estimated positional accuracy of approximately 2 cm. In contrast, total station measurements can be assumed to have an accuracy of within 5 cm under field conditions. Reference points were established using GNSS, and detailed measurements of the area, streambed, and structures were taken using a total station. This served as the baseline/reference for accuracy comparison (red points in Figure 2). Using a combination of GNSS measurements and tacheometry, both planimetric and altimetric features of the streambed and its structures were surveyed, and reference points were subsequently measured for accuracy comparison. First, the entire streambed (217 points) and retention structures (116 points) were surveyed, followed by the survey of nine reference points on the forest road, an additional 43 reference points within the profiles, and 45 points for scan merging and subsequent georeferencing (Figure 2).

The LiDAR scanning was performed using an iPhone 14 Pro (Apple Inc., Cupertine, CA, USA) with a LiDAR sensor, as enabled by the 3D Scanner App (Laan Labs Consulting, NY, USA) [33]. The iPhone LiDAR sensor operates on a triangulation-based principle, combining infrared laser emission with reflected signal imaging, which allows for high-resolution depth mapping at short ranges. The iPhone 14 Pro uses a LiDAR scanner based on a VCSEL array, which emits thousands of infrared pulses per second. The depth map resolution is approximately 256 × 192 pixels, resulting in around 49,000 depth points per frame, with a scanning frame rate of up to 60 Hz, depending on the application [45]. The calibration between the LiDAR sensor and the RGB camera is handled internally by the scanning application, which fuses depth and image data in real time using built-in algorithms. This fusion enables synchronized acquisition of spatial and visual information, enhancing the usability of the resulting 3D models [46]. Existing studies [38] indicate that devices (and the models they create) achieve excellent accuracy at shorter distances, but errors accumulate, and accuracy decreases as the distance increases. Additionally, accuracy is significantly negatively affected by changes in the movement direction during scanning, leading to angular errors. The speed of movement influences the density of the scanned point cloud during data acquisition; in our case, the average point density was approximately 8000 points per square meter, assuming a steady scanning pace and optimal device orientation.

**Figure 3:**
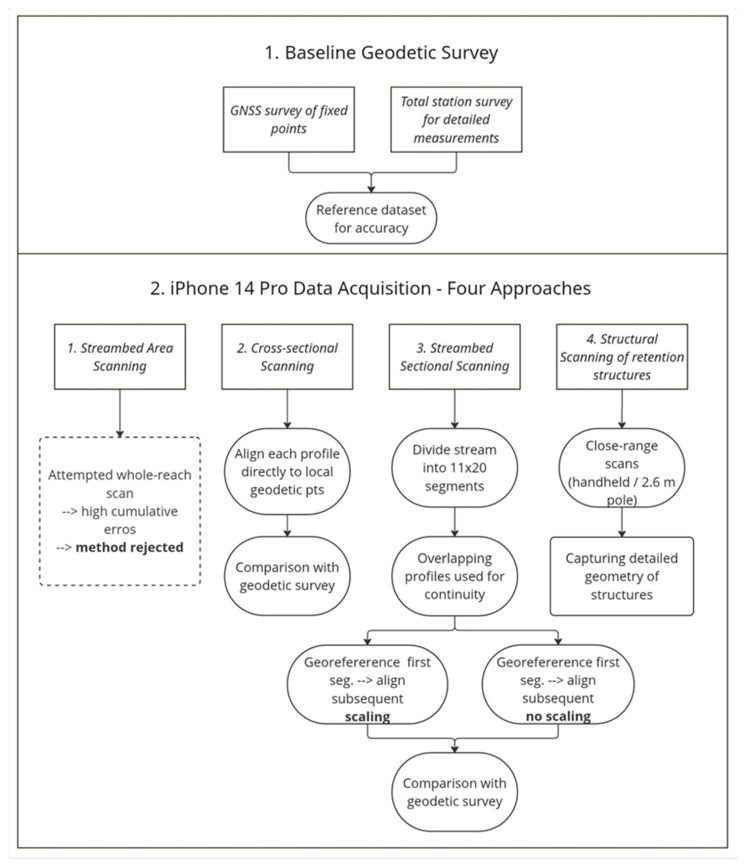
Workflow diagram illustrating baseline geodetic survey and four iPhone 14 Pro LiDAR acquisition approaches, followed by data processing and alignment for streambed and structure mapping in forested terrain.

Four scanning and subsequent data evaluation procedures were utilized, considering the findings above (Figure 3). Specifically, these included streambed area scanning, cross-sectional scanning, sectional area scanning, and scanning of retention structures. For streambed and forest road scanning, the device was handheld, and the operator moved slowly in a specified direction, maintaining a consistent scanning angle and pace. The iPhone was manually rotated to capture the surrounding geometry, while the scanning process was monitored in real-time via the display. For retention structures and hard-to-reach areas, the iPhone was mounted on a 2.6 m geodetic telescopic pole, allowing for safe and stable scanning from above. The vertical orientation of the device was preserved by using visual alignment and consistent hand positioning.

#### 2.2.1. Streambed Area Scanning Method

The initial aim was to perform area scanning of the streambed, including structures on the stream, with subsequent comparison of positional and elevation accuracy based on reference points surveyed with a total station or a GNSS device. Metal and plastic reference points were chosen as reference markers, as they would be easily identifiable on the scanned model for precise position determination. These points were regularly distributed throughout the streambed and on the banks, and were surveyed both in terms of position and elevation.

The initial scanning was performed to capture the entire streambed at once by moving in all directions within the streambed. However, issues arose with RAM capacity during scanning, data processing, and model coloring, leading to the application frequently becoming unresponsive. When scanning a large area, the device undergoes frequent rotation, accumulating positional and altitude errors. Significant positional deviations, ranging from tens of centimeters to meters, were discovered. Higher accuracy was achieved when scanning against the direction of movement, but overall, this method was deemed unsuitable and thus not included in the study’s results.

#### 2.2.2. Cross-Sectional Scanning Method

**Figure 4:**
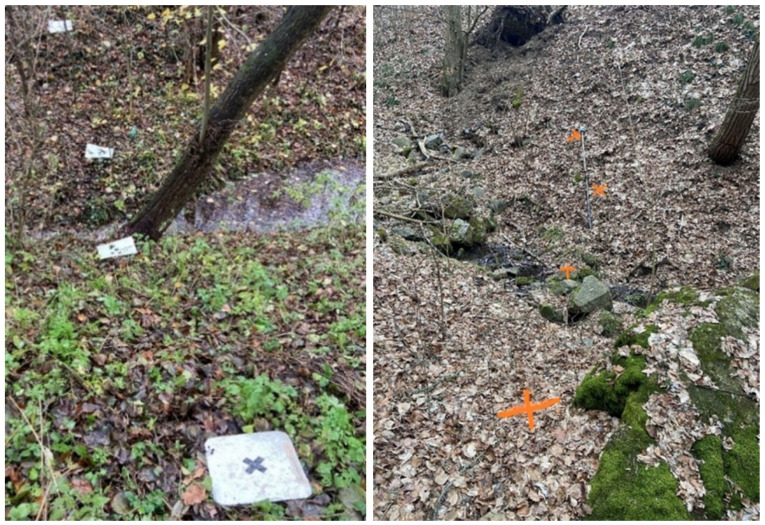
(**Left**)—Cross profile with marked reference points (plastic and metal target), (**Right**)—spray painted markers for sectional scanning (orange spray paint).

In subsequent measurements, reference points in the form of metal and plastic targets were first placed on the body of the adjacent forest road and at nine cross-sections on the stream (Figure 4). Nine points on the forest road (yellow crosses in Figure 2) and five points at terrain break lines were marked in each profile (yellow triangles in Figure 2). These points were again surveyed using GNSS and a total station.

**Figure 5:**
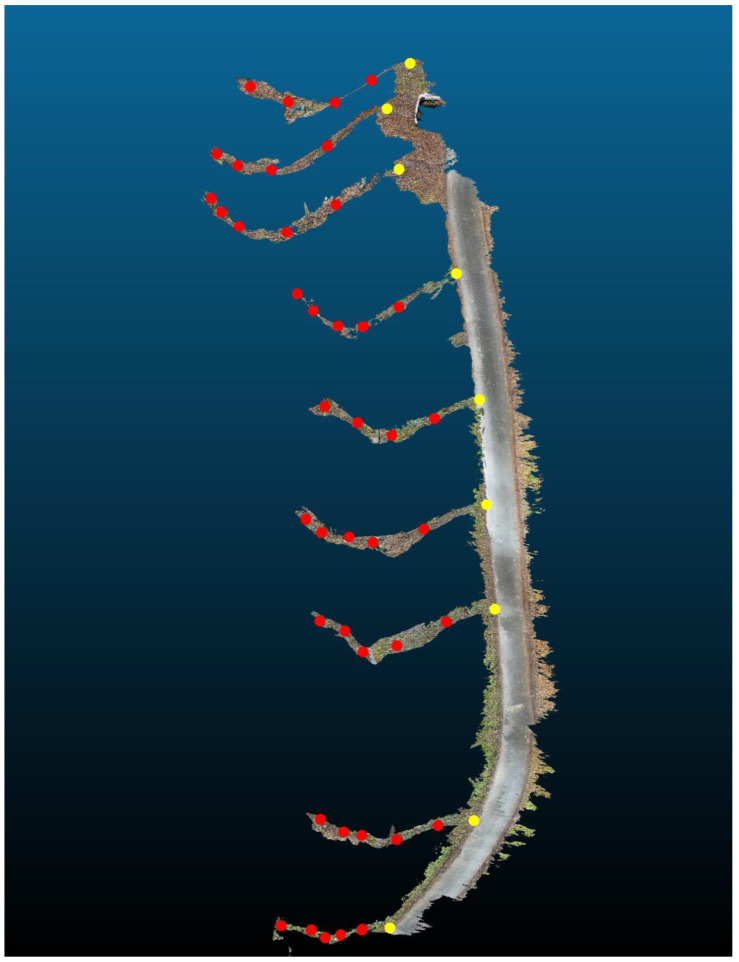
Cross-sectional scanning (control points on forest road—yellow, control points in profiles—red).

To improve the alignment of individual scans, the scanning was consistently performed against the direction of movement. This approach was based on prior experience indicating that scanning in the opposite direction to walking or stream flow reduces cumulative errors. When moving forward while scanning, frequent device rotations and changes in scanning angle can introduce distortions in the point cloud geometry. In contrast, scanning backward allows for more stable device orientation and smoother data acquisition, which facilitates better overlap between adjacent scans. Initially, nine reference points were scanned along the adjoining forest road in a longitudinal direction. These points served as fixed anchors for aligning subsequent scans. Following this, individual cross-sectional profiles of the streambed were scanned. Each profile was captured in such a way that it included at least three of the previously scanned reference points on the forest road. This ensured sufficient overlap for accurate alignment of the scans using georeferencing techniques. The consistent inclusion of known reference points in each scan significantly improved the spatial continuity and positional accuracy of the resulting 3D model. As a result, the alignment of individual sections becomes more accurate and less prone to drift, especially in complex terrain such as forested streambeds (Figure 5).

#### 2.2.3. Streambed Sectional Scanning Method

**Figure 6:**
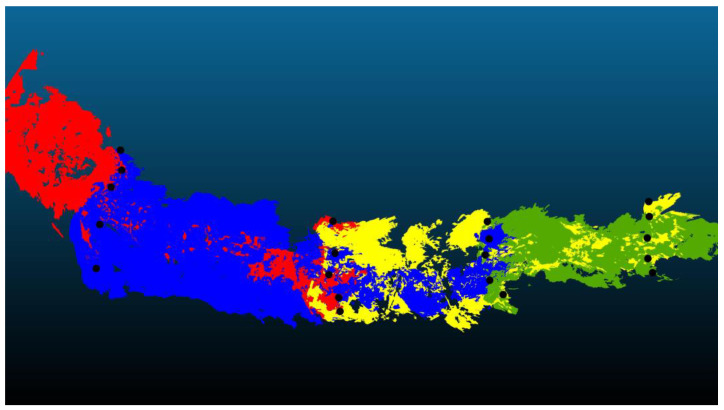
Alignment of several overlapping sections (each scanned section differentiated in color, e.g., red, blue, yellow, and green—four overlapping sections, with control points in black color).

The entire streambed was divided into 11 segments, each approximately 20 m long. Within each segment, three cross-sectional profiles were created, typically consisting of 4 to 5 control points. All control points in these profiles were geodetically surveyed using GNSS and a total station. In the first segment, the first two profiles were used for georeferencing the scanned data. In subsequent segments, scanning was performed with intentional overlap: each new segment included two profiles from the previous segment and one new profile. These overlapping profiles enabled alignment of the scans relative to each other (Figure 6). The control points in the overlapping profiles were not used for georeferencing but served as independent checkpoints for evaluating positional and elevation accuracy. This approach ensured continuity of the streambed model while allowing for a detailed assessment of error propagation across the entire scanned area. Due to the large number of points, the individual control points were marked only with spray paint on the terrain or stones in the streambed, unlike the previous method (Figure 4).

#### 2.2.4. Structural Scanning of Retention Structures on the Streambed Method

Three retention structures within the section under consideration have been constructed on the Dílský stream. These retention structures were surveyed using a total station first and an iPhone 14 Pro with a LiDAR sensor. The stationing of the structures within the surveyed section is as follows: the first retention structure is located at 45.47 m, the second retention structure at 117.57 m, and the last retention structure, which also marks the end of the surveyed section, at 189.29 m (Figure 1).

The first retention structure is constructed from a combination of stone, concrete, and iron, while the remaining two are made of plain stone masonry combined with concrete. Water is conveyed through a perforated pipe at the second retention structure, while at the last retention structure, water is transferred through two seepage holes created in the structure’s body. The overflow edges of the structures also differ: the first retention structure has a rectangular overflow, while the remaining two have trapezoidal overflows.

The scanning was performed by moving along the retention structures and tilting and rotating the device to ensure complete capture of the objects and creating a continuous model.

### 2.3. Data Processing and Analysis

The workflow for processing and analyzing LiDAR data collected with an iPhone 14 Pro involved alignment, georeferencing, vectorization, and accuracy evaluation. The software used included ArcGIS Pro (Version 3.2, Esri, Redlands, CA, USA) [47], CloudCompare (Version 2.14, EDF R&D, Paris, France) [48], AutoCAD (Version 24.3, Autodesk Inc., San Rafael, CA, USA) [49], and the 3D Scanner app (Version 5.1, Laan Labs, Amsterdam, The Netherlands) [33]. Four methods (streambed area scanning, cross-sectional scanning, streambed sectional scanning, and scanning of structural objects) were employed to ensure comprehensive data collection. Initially, the 3D scans were exported from the 3D Scanner App as OBJ files and imported into CloudCompare. Each scan represented a portion of the study area, and these segments were sequentially aligned and merged to create a complete model.

Individual scans were aligned using the Align tool in Cloud Compare software (v2.14) to ensure spatial continuity, matching corresponding points across overlapping scans. Following alignment, the model was georeferenced with geodetic points collected by GNSS and total station surveys. This process adhered to the S-JTSK coordinate system, ensuring that the LiDAR data could be accurately compared to the traditional survey data.

In the case of cross-sectional scanning, the entire scan of the forest road was aligned to the first three reference points, and the remaining six geodetically measured points along the road were used as independent checkpoints to assess positional and vertical accuracy. This allowed us to quantify the deviation between the LiDAR-derived coordinates and the geodetic measurements, providing a reliable estimate of scanning precision over the scanned segment. Because positional and vertical errors increase significantly with scanning distance, aligning cross-sectional scans to previously georeferenced segments proved ineffective due to the cumulative nature of these deviations. Therefore, in the second procedure, each cross-sectional profile was aligned directly to its corresponding geodetically surveyed control points. This approach minimized error propagation and ensured higher local accuracy.

For the streambed sectional scanning, the first scanned segment was georeferenced using geodetically surveyed control points from the first two cross-sectional profiles. These points were measured using GNSS and a total station, serving as the spatial anchor for the initial alignment. All subsequent segments were scanned with intentional overlap. Instead of using additional geodetic anchoring, they were aligned relative to each other based on identifiable control points visible in the overlapping scans. These overlapping points were not used for georeferencing but served as independent checkpoints for evaluating positional and vertical accuracy. This approach enabled the creation of a continuous 3D model of the streambed, allowing us to monitor cumulative deviations over distance and assess the reliability of scan alignment without requiring repeated geodetic input. In the case of scanning retention structures on the streambed, no georeferencing was performed on the outputs. Instead, the evaluation focused solely on comparing the dimensions of the structures derived from the 3D mesh models with those obtained through geodetic measurements using a total station. Measurements, such as width, height, and length of structural elements, were extracted from the LiDAR models and compared to their corresponding geodetic values. Deviations were assessed based on identifiable edges and contours, without relying on coordinate-based alignment. This approach allowed for a practical assessment of the iPhone LiDAR’s capability to capture object geometry under field conditions, while acknowledging limitations due to vegetation occlusion and material reflectivity.

The final georeferenced models required structuring for further analysis. Using CloudCompare’s Point List Picking tool, reference points were vectorized from models to create a shapefile with X, Y, and Z values. This vectorized data enabled seamless integration with GIS software (ArcGIS Pro, v3.2), facilitating further spatial analysis. ArcGIS Pro was used for map creation, and AutoCAD (v24.3) was used for drawing preparation.

The final step involved assessing the accuracy of the mobile LiDAR data by comparing it with geodetic reference points. Positional deviations in the XY plane and elevation differences (*Z*-axis) were calculated. The accuracy was quantified using the RMSE, which averaged the squared differences between corresponding points to provide an overall measure of alignment accuracy.

## 3. Results

The primary objective of this part of the study was to evaluate the positional and vertical accuracy of mobile LiDAR scanning by comparing the scanned data with geodetically surveyed control points. These control points served as a reliable reference for assessing deviations in both horizontal and vertical dimensions. By analyzing the differences between the LiDAR-derived coordinates and the geodetic measurements, we aimed to quantify the precision of the iPhone 14 Pro LiDAR sensor under real-world conditions and determine its suitability for mapping small-scale landscape features.

### 3.1. Cross-Sectional Scanning

**Figure 7:**
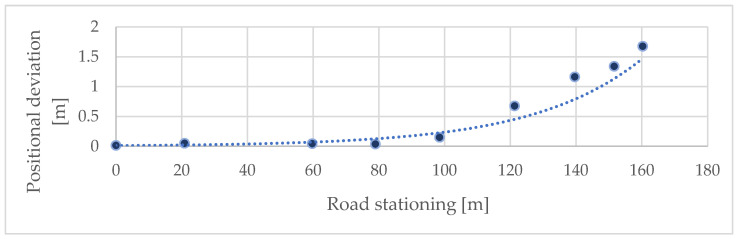
Effect of distance on positional accuracy (control points on the forest road).

**Figure 8:**
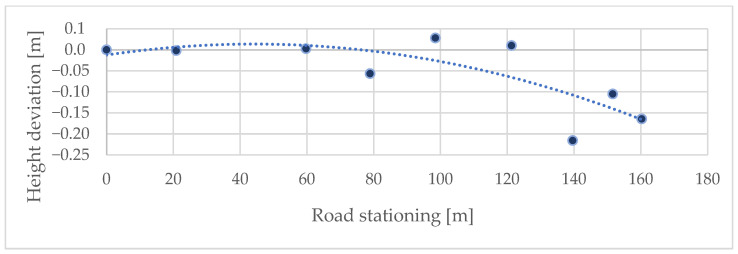
Effect of distance on height accuracy (control points on the forest road).

The comparison was made between the coordinates obtained from total station measurements and those extracted from the co-registered iPhone LiDAR point cloud. The initial evaluation focused on determining the positional and height accuracy of the LiDAR data captured by the iPhone 14 Pro on the forest road (or close to the forest road), where the horizontal deviation was 0.571 m, with a standard deviation of 0.623 m. The root mean square error (RMSE) for these measurements was calculated to be 0.845 m. These results indicate that the scanning distance significantly affects mobile LiDAR accuracy, with reliable accuracy up to 60–70 m. Beyond this range, the errors increased substantially. The height accuracy of the measured points is slightly better. Additionally, minor positional and elevation deviations at the beginning of the section are due to georeferencing the model to the first three reference points (Figure 7 and Figure 8). The comparison of position and height accuracy according to the above-mentioned statistical variables is shown in Table 1 (Forest Road column). It shows that the positional error increases with distance; however, the height error is only slightly affected.

Aligning the scanned cross-sectional profiles to the georeferenced road segment proved unsuitable, as it led to the accumulation of positional and vertical errors with increasing scanning distance. Therefore, instead of relying on the previously aligned road scan, each cross-sectional profile was independently aligned directly to its corresponding geodetic control points. This approach significantly improved accuracy, especially over short distances, where positional and vertical RMSE values of up to 0.12 m were achieved (Table 1, Cross profiles column).

### 3.2. Streambed Scanning

**Figure 9:**
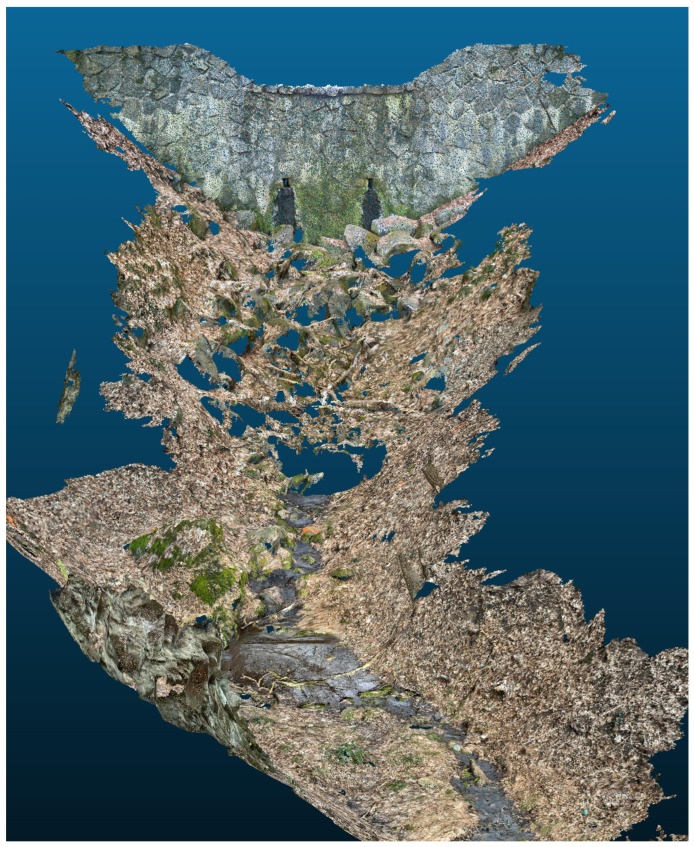
D reconstruction of the upper section: Merged photogrammetric scans showing structural continuity and surface detail of the streambed and embankments.

Creating a continuous 3D model of the stream bed required integrating several individual scans and testing various alignment and scaling techniques. This involved a two-part procedure: (i) the initial integration of scans without scaling adjustments, and (ii) integration with scaling adjustments to enhance accuracy. The aligned scans were then merged into a continuous model of the streambed (Figure 9). The coordinates of the control points from the merged models were then vectorized and compared with the surveying measurements.

Initially, each scan was integrated without any scale modification—the individual scans were connected by aligning overlapping control points in each profile to create a continuous model. This direct alignment resulted in notable inaccuracies, including positional and height misalignments (Table 2, No scale column).

In this context, ‘scaling’ refers to adjusting the LiDAR scan dimensions to match the scale of geodetically measured reference points, thereby compensating for distortions caused by device movement or environmental factors. Scaling adjustments in the CloudCompare Align tool were applied to each profile to address these misalignments. Scaling involved calibrating the scale of each LiDAR scan relative to reference points obtained from geodetic measurements (in the case of the first scan, for subsequent scans, the scale was adjusted based on the previous scan), allowing for proportional correction across all profiles (Table 2, Scale column).

**Figure 10:**
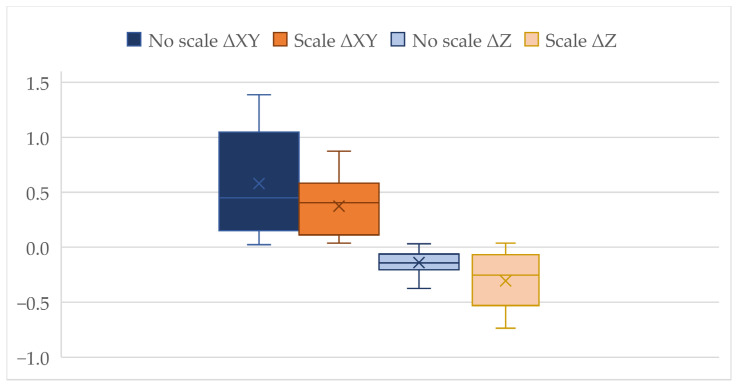
Distribution of positional (ΔXY) and vertical (ΔZ) errors for streambed sectional scans with and without scaling.

Scale modifications influence positional and vertical accuracy. Applying scaling during the alignment of scans increased the positional accuracy of the resulting model, with the positional RMSE decreasing from 0.760 m (No scale) to 0.449 m (Scale). The altitude RMSE increased from 0.172 m (No Scale) to 0.436 m (Scale), which is consistent with the stepwise alignment approach used in this study. Since each scan was georeferenced independently, rather than as part of a unified block adjustment, local distortions in elevation were not fully compensated during the scaling process, resulting in increased vertical deviations, see Figure 10.

The boxplots show the error distributions for horizontal (ΔXY), and vertical (ΔZ) misalignments derived from control point comparisons. Dark blue and dark orange boxplots represent positional errors without and with scaling, respectively, while light blue and light orange indicate vertical errors without and with scaling. In each dataset, the box edges mark the interquartile range, the central line indicates the median, the ‘X’ marker denotes the mean, and whiskers represent the minimum and maximum values. Scaling reduces horizontal deviations but increases vertical errors, consistent with the RMSE values in Table 2.

**Figure 11:**
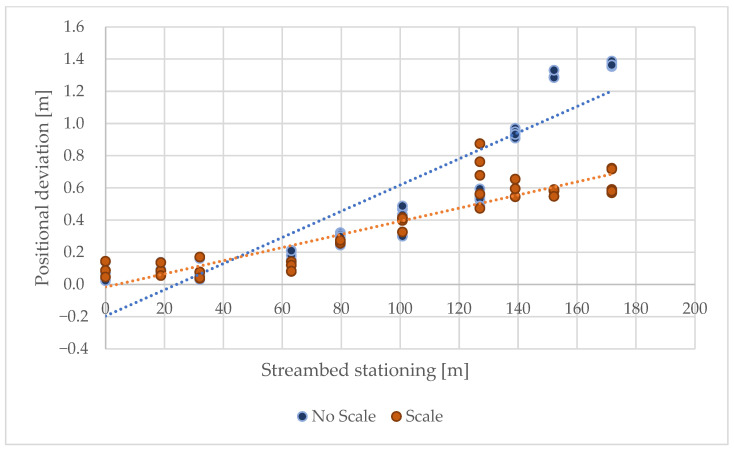
Dependence of positional deviation on scanning distance.

**Figure 12:**
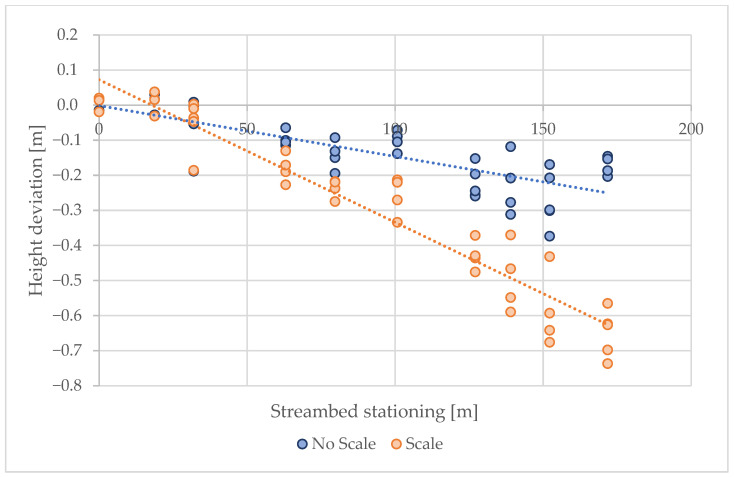
Dependence of height deviation on scanning distance.

Similarly to the control points on the forest road, the influence of distance on the resulting positional and altitude accuracy was also evaluated, with the difference being that the RMSE of all control points in the individual profiles was assessed separately. It was again demonstrated that deviations significantly increase with distance. Without surveying the control points, this scanning method can be used for distances up to 60 m (Figure 11 and Figure 12).

Positional deviations increase with streambed stationing, indicating an apparent distance-related decline in accuracy. The application of scaling substantially reduces horizontal misalignments compared to the unscaled approach, as reflected in both the magnitude of deviations and the slope of the linear trend. This demonstrated that scaling provides more consistent positional accuracy over longer scanning distances.

Height deviations also increase with distance from control points, but the effect differs between scaling and non-scaling approaches. Without scaling, deviations remain relatively stable around the zero baseline with only minor negative drift. By contrast, scaling introduces a systematic negative bias, with vertical errors becoming progressively larger at greater distances. This highlights a trade-off between improved positional accuracy and decreased vertical consistency when scaling is applied.

### 3.3. Streambed Sectional Scanning—Georeferencing Sections to All Reference Points

To address the positional and vertical deviations observed in previous scanning methods, a refined approach was applied in the form of sectional scanning combined with geodetic georeferencing. In this method, the streambed was divided into segments approximately 20 m long. Each segment was individually georeferenced using geodetically surveyed control points measured by GNSS and a total station. Unlike the previous method, where only the first segment was georeferenced and subsequent segments were aligned based on overlapping scan features, each segment in this method was anchored directly to its own set of geodetic reference points. This ensured consistent spatial accuracy across the entire scanned area and minimized cumulative errors.

Further validation was performed using terrain points outside the reference set (217 terrain points, red dots in Figure 2). This analysis yielded an RMSE of 0.16 m (Table 3). The most significant deviations (up to 0.75 m) were observed at the edges of the scanned area, likely due to interpolation artifacts. These results confirm that reliable vertical accuracy can be achieved by combining mobile LiDAR scanning with geodetic reference points spaced approximately every 20 m. Although this method requires geodetic surveying of control points, the integration with iPhone LiDAR scanning enables the acquisition of dense and spatially continuous surface data, which provides significantly higher detail than traditional geodetic measurements alone. This combination allows for efficient terrain modeling in complex environments, where conventional methods are hindered by time, cost, or accessibility limitations.

### 3.4. Performance in Mapping Small Water Structures

**Figure 13:**
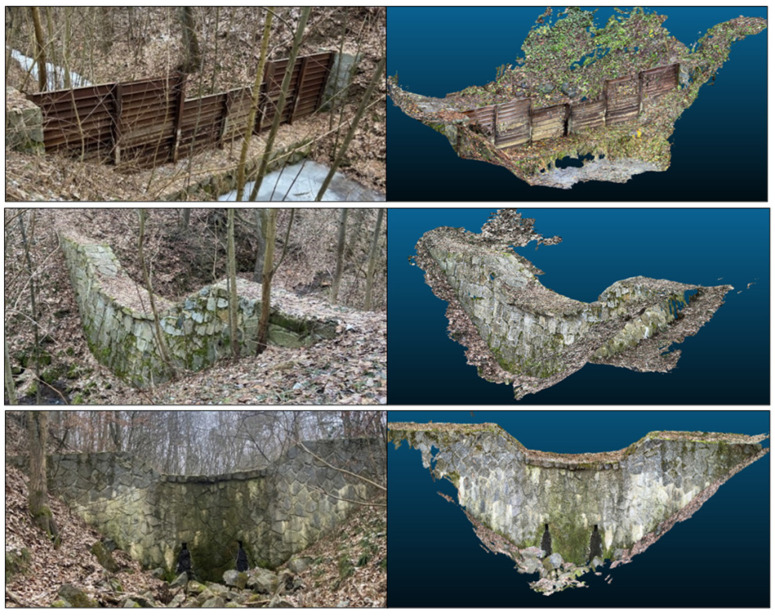
The addressed retention structures on the streambed with associated mesh models—(**top**) the first retention structure (a combination of stone masonry with concrete and iron), (**middle**) the second retention structure (a combination of stone masonry with concrete), (**bottom**) the third retention structure (a combination of stone and concrete).

Mapping small water retention structures presented mixed results. Due to the nature of the data, where LiDAR-derived models and geodetic measurements do not share identical point locations, standard RMSE calculations were not directly applicable. Instead of interpolating LiDAR data to geodetic points, deviations were assessed by comparing identifiable structural features—such as edges, corners, and dimensions—between the LiDAR-derived mesh models and total station measurements. The iPhone 14 Pro’s LiDAR sensor effectively captured essential structural features in clear, unobstructed areas. For three distinct retention structures along the stream (Figure 13), the mean positional accuracy was within a 7.0% margin of error compared to geodetic measurements. Detailed comparisons of individual dimensions, including percentage deviations, are provided in Supplementary Materials S1–S3. However, in areas where the structures had complex geometries (e.g., stone-metal composites) or where visibility was obstructed by vegetation, positional errors up to 20 cm were observed. The highest deviations were recorded in narrow parts (e.g., the width of the iron overflow at retention structure No. 1) and at the connection of the retention structure to the terrain, where part of the structure was covered with leaves, making it impossible to determine the edge on the scan. These results highlight the sensitivity of mobile LiDAR to changes in material reflectivity and scan occlusions. Despite these limitations, the iPhone 14 Pro LiDAR sensor demonstrated effective mapping capabilities for small water structures under suitable conditions.

## 4. Discussion

The study results demonstrate that LiDAR in smartphone devices achieves excellent output quality and accuracy when scanning smaller objects and terrain at shorter distances of up to 20 m (or up to 60 m with an error of 20 cm). This is consistent with studies that highlight the sensitivity of mobile LiDAR systems to canopy structure and terrain slope [50,51].

The rationale behind selecting and comparing the four scanning methods—area, cross-sectional, sectional, and structural—was to systematically assess the strengths and limitations of the iPhone LiDAR sensor under various field conditions. Area scanning was initially considered for its potential efficiency in capturing large sections at once; however, practical limitations, such as device memory and error accumulation, rendered it unsuitable for detailed mapping. Cross-sectional scanning provided high local accuracy and was effective for validating LiDAR data against geodetic reference points, though it lacked continuous coverage. Sectional scanning offered a compromise, enabling the creation of a constant model with reduced cumulative errors by dividing the stream into shorter, georeferenced segments. Structural scanning was essential for capturing the detailed geometry of small water retention structures, but its effectiveness was sometimes limited by accessibility and vegetation. This comparative approach allowed us to identify optimal workflows for different mapping scenarios and highlighted the trade-offs between efficiency, accuracy, and field practicality.

It has been proven that merging scans improves the final positional and height accuracy of the model; however, it also leads to the accumulation of errors. In the results, a noticeable difference was observed between vertical (Z) and horizontal (XY) accuracy. The vertical deviations were significantly smaller than the horizontal ones. This can be attributed to the nature of the iPhone LiDAR sensor, which measures depth directly using time-of-flight technology, resulting in relatively stable elevation data. In contrast, horizontal positioning relies on device movement and orientation, which introduces cumulative errors, especially during large-area scanning with frequent rotations.

The scaling method can partially eliminate these errors, but it is still impossible to map over longer distances without geodetically surveyed reference points. The vertical error observed when using the scaling method is primarily caused by the uniform scaling of point clouds, which does not fully account for localized distortions in elevation due to device movement or terrain complexity. Using the sectional scanning method with georeferencing to all reference points resulted in a vertical RMSE of 0.16 m. This suggests that with properly spaced geodetic reference points (every 20 m), mobile LiDAR can provide reliable vertical accuracy for streambed modeling. However, positional errors tended to accumulate with increasing scanning distance, limiting the standalone applicability of mobile LiDAR in large-scale mapping tasks. It is possible to use direct scanning without reference points in cases where small construction objects are located on watercourses, such as retention structures. The output can then be directly vectorized into project documentation. However, small details up to 10 cm in size may be captured with less accuracy, so manual measurement is recommended. The advantage is capturing the overall condition of the objects.

The positional and vertical accuracy achieved in this study using iPhone 14 Pro LiDAR is consistent with recent comparative research. For example, authors in their study [37] found that iPhone LiDAR is capable of capturing stream channel morphology with millimeter to centimeter accuracy, although its performance decreases for finer features. The following study [36] identified mean absolute errors of around 1 cm in controlled terrain change detection; however, accuracy declined in complex environments. In forestry, authors in a study [38] reported RMSEs for planimetric and vertical accuracy at 0.185 m and 0.021 m, respectively, when using iPhone LiDAR for forest road profiling, showing slightly higher errors than dedicated terrestrial or personal laser scanners, but sufficient for rapid field surveys. These results confirm that while iPhone LiDAR cannot replace high-end surveying in all environments, it offers a competitive and low-cost alternative for detailed mapping and monitoring, particularly where high portability and efficiency are top priorities.

When comparing mobile LiDAR measurements with traditional GNSS and total station surveys, the iPhone LiDAR sensor showed comparable accuracy in open, vegetation-free areas. However, the accuracy diminished significantly in sections with dense canopy cover or steep slopes. In such conditions, the horizontal errors exceeded 1 m, making the mobile LiDAR unsuitable as a standalone solution for precise surveying. Nevertheless, using reference points from geodetic surveys significantly improved the alignment and reliability of the LiDAR-based models. The influence of environmental factors, such as dense canopy cover, on LiDAR accuracy also aligns with findings from other studies, which suggest that combining LiDAR with alternative remote sensing techniques, such as UAV-based photogrammetry, can provide a more comprehensive solution for environmental monitoring [52,53].

In practical applications, integrating mobile LiDAR with traditional geodetic tools, such as GNSS or total stations, can significantly enhance accuracy, particularly for stream bed profiling or when mapping small water structures with complex geometries. The study’s results indicate that post-processing adjustments, such as scaling and alignment, are crucial to mitigate the deviations observed in raw LiDAR data. This approach is supported by the research of [14], which emphasizes the combination of terrestrial laser scanning (TLS) or mobile platforms with additional reference points to achieve reliable results for detailed terrain models. Authors in this study [40] evaluated the Apple iPhone 12 Pro LiDAR for terrain change detection and found it capable of producing accurate 3D models with an absolute accuracy of ±1 cm. Similarly, study [25] explored its use in rapid 3D mapping for indoor and outdoor environments, emphasizing cost efficiency while acknowledging limitations in large-scale reconstructions.

Several studies highlight iPhone LiDAR’s potential in environmental and forestry applications. Authors of this study [54] assessed its use for urban forest inventories. They compared its accuracy with terrestrial laser scanning and found it to be a viable alternative for tree diameter measurements, yielding RMSE values of 1.74–3.30 cm. Integration with augmented reality was demonstrated for improved efficiency in forest inventory assessments [55], where the RMSE_DBH_ varied between 1.15 and 1.92 cm, while [36] tested it for estimating single-tree parameters, concluding that while practical (DBH measurement RMSE 3.13 cm), it lacks the precision of high-end laser scanning methods. Additionally, ref. [38] explored its use in assessing forest road damage, finding that while iPhone LiDAR provides valuable insights, its accuracy remains lower than terrestrial laser scanning for detailed condition monitoring. Their study achieved positional height accuracy compared to tachymetric surveying using an iPhone 13 Pro with devXY and devZ RMSE of 0.185 m and 0.021 m, respectively.

In agricultural studies, authors [56] found strong correlations between iPhone LiDAR and photogrammetric methods when measuring surface roughness in farmlands, demonstrating the cost-effectiveness and efficiency of LiDAR for soil evaluation. In a study [36], authors have further explored its potential for high-resolution soil erosion mapping, suggesting that while it performs well in localized assessments, larger-scale studies require integration with UAV photogrammetry or GNSS data. Beyond terrestrial applications, ref. [57] investigated the integration of iPhone LiDAR with UAV photogrammetry for sinkhole hazard mitigation, emphasizing its benefits in detecting subsurface cavities and assessing geohazards. Similarly, ref. [58] demonstrated its effectiveness in coastal geomorphology, particularly in mapping sea caves and related landforms, reinforcing its value in marine topographic studies.

Future work should focus on refining smartphone LiDAR processing algorithms and exploring hybrid methods that integrate data from multiple platforms to improve reliability and extend their applicability in complex environments. A similar study [37] has demonstrated the potential of iPhone LiDAR for capturing small-scale geomorphic and topographic features, particularly under controlled conditions. However, these studies also highlight their limitations in dense vegetation and over extended distances, where positional and height accuracy errors become significant. Similarly, authors [59] emphasized the importance of post-processing techniques, such as scaling and alignment, to mitigate inaccuracies in more complex environments. Additionally, it is worth noting that this study did not address the correction of LiDAR point cloud intensity distortions caused by the distance and incidence angle effects. Prior research has highlighted that uncorrected intensity values may misrepresent the actual echo intensity characteristics of natural surfaces, such as streambeds and small water bodies in forested terrain [60,61]. Intensity correction methods thus improve the physical accuracy and consistency of reflectance measurements. Incorporating such radiometric corrections in future studies could significantly enhance the utility of mobile LiDAR data for detailed environmental characterization, complementing the geometric accuracy assessments presented here. Collectively, these findings align with our results, underscoring that while iPhone LiDAR represents a promising and accessible tool for preliminary geospatial data collection, integrating it with traditional geodetic methods or advanced post-processing workflows remains critical for achieving optimal accuracy and reliability in diverse terrains. Recent advancements in SLAM (Simultaneous Localization and Mapping) applications hold promise for enhancing mobile LiDAR scanning capabilities using smartphones. Tools such as RTAB-Map and DepthViz enable real-time mapping and pose estimation without relying on GNSS, which is particularly beneficial in forested or GNSS-limited environments. RTAB-Map supports loop closure detection and multi-session mapping, while DepthViz integrates LiDAR, camera, and IMU data for enhanced spatial accuracy. Additionally, AI-based post-processing techniques can correct motion-induced distortions, interpolate missing data caused by vegetation occlusion, and automate the merging of scans. RTAB-Map has primarily been applied in robotics and autonomous navigation, where its SLAM capabilities have been extensively validated across various datasets and platforms. However, its use in environmental studies, particularly with mobile devices such as the iPhone, remains largely unexplored. This gap presents an opportunity for future research to evaluate RTAB-Map’s potential in outdoor environmental mapping using smartphone-based LiDAR systems [62]. These technologies significantly enhance the reliability and usability of iPhone LiDAR data for environmental mapping and should be considered in future research.

## 5. Conclusions

This study demonstrates that iPhone 14 Pro LiDAR sensors, when used under optimal conditions, can provide a rapid and relatively accurate method for preliminary surveying small-scale water structures and stream sections. Its strengths lie in its portability, ease of use, and capacity to generate high-resolution 3D models quickly, making it a valuable tool for environmental monitoring, particularly in small or moderately complex areas. However, the limitations observed, such as increased errors in dense vegetation and decreased vertical accuracy in complex terrains, suggest that iPhone 14 Pro LiDAR is not a viable replacement for traditional surveying methods in all contexts.

Combining smartphone LiDAR with traditional geodetic tools is recommended for practical applications to achieve optimal accuracy. Structured scanning methodologies—including control points, overlapping scans, and post-processing adjustments—are essential for minimizing errors and improving data quality. The vertical accuracy achieved through the sectional scanning method confirms the potential of smartphone LiDAR for reliable streambed modeling when combined with adequately spaced reference points. This combined approach can compensate for the weakness of individual methods and provide a comprehensive toolkit for environmental management. As noted in studies [14,51], mobile and airborne LiDAR hybrids can produce detailed and accurate models, even in complex landscapes.

For broader adoption, future research should prioritize the development of advanced processing algorithms, such as SLAM, that address the challenges of terrain conclusion and data fusion and explore the potential of lightweight and cost-effective scanning systems for use in remote and forested regions. Such advancements would significantly expand the practical utility of smartphone LiDAR for environmental monitoring and geospatial data collection in challenging settings. Ongoing research is expected to address these limitations, paving the way for the adoption of broader hydrological and ecological studies.

## Figures and Tables

**Table 1:** Evaluation of cross-sectional scanning.

Cross-Sectional Scanning	Forest Road		Cross Profiles	
	Δxy [m]	Δz [m]	Δxy [m]	Δz [m]
Mean	0.571	−0.056	0.115	0.016
StD	0.623	0.082	0.068	0.109
Max	1.675	0.028	0.308	0.326
Min	0.014	−0.216	0.008	−0.176
RMSE	0.845	0.099	0.115	0.110

**Table 2:** Evaluation of streambed sectional scanning.

Streambed Scanning	No Scale		Scale	
	Δxy [m]	Δz [m]	Δxy [m]	Δz [m]
Mean	0.580	−0.139	0.374	−0.311
StD	0.491	0.101	0.248	0.305
Max	1.387	0.031	0.875	0.482
Min	0.023	−0.374	0.038	−1.334
RMSE	0.760	0.172	0.449	0.436

**Table 3:** Evaluation of height accuracy in the combination of geodetic reference points and scanning.

A Combination of Geodetic Reference Points and Scanning	Δz [m]
Mean	−0.05
StD	00.15
Max	0.56
Min	−0.75
RMSE	0.16

## Data Availability

The data that support the findings of this study are available from the corresponding author, upon reasonable request.

## Supplementary Materials

The following supporting information can be downloaded at: https://www.mdpi.com/article/10.3390/s25196141/s1. Supplementary Materials S1–S3 contain detailed comparisons of measurements of individual retention structures. These materials are published online alongside the manuscript.
