# Supplementary material for: Accuracy Assessment of iPhone LiDAR for Mapping Streambeds and Small Water Structures in Forested Terrain
Source: Sensors (Basel). 2025 Oct 4;25(19):6141. doi: 10.3390/s25196141 (PMC12526706; doi:10.3390/s25196141)
Supplement: Supplementary file 1 [file sensors-25-06141-s001.zip › S1_structure_1.pdf]

SECTION A - A´

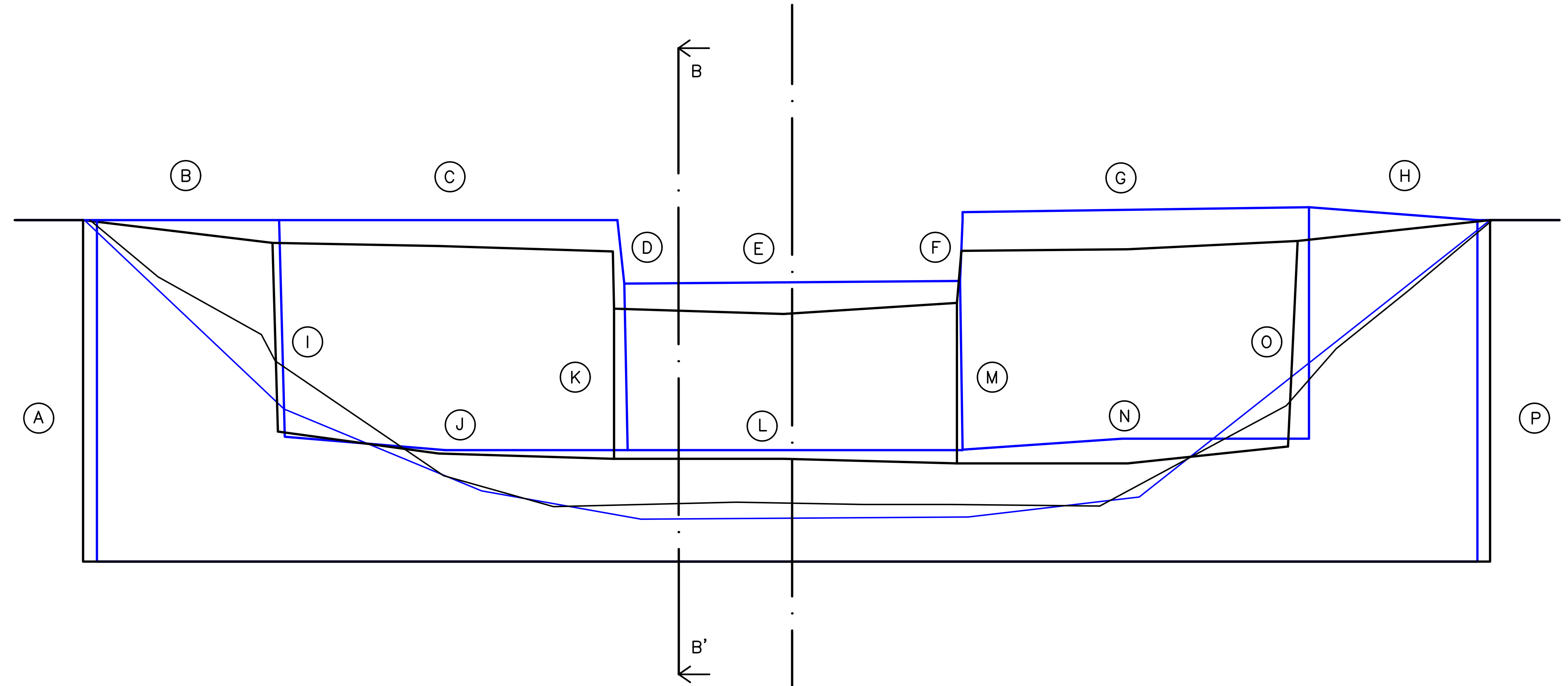

SECTION B - B´

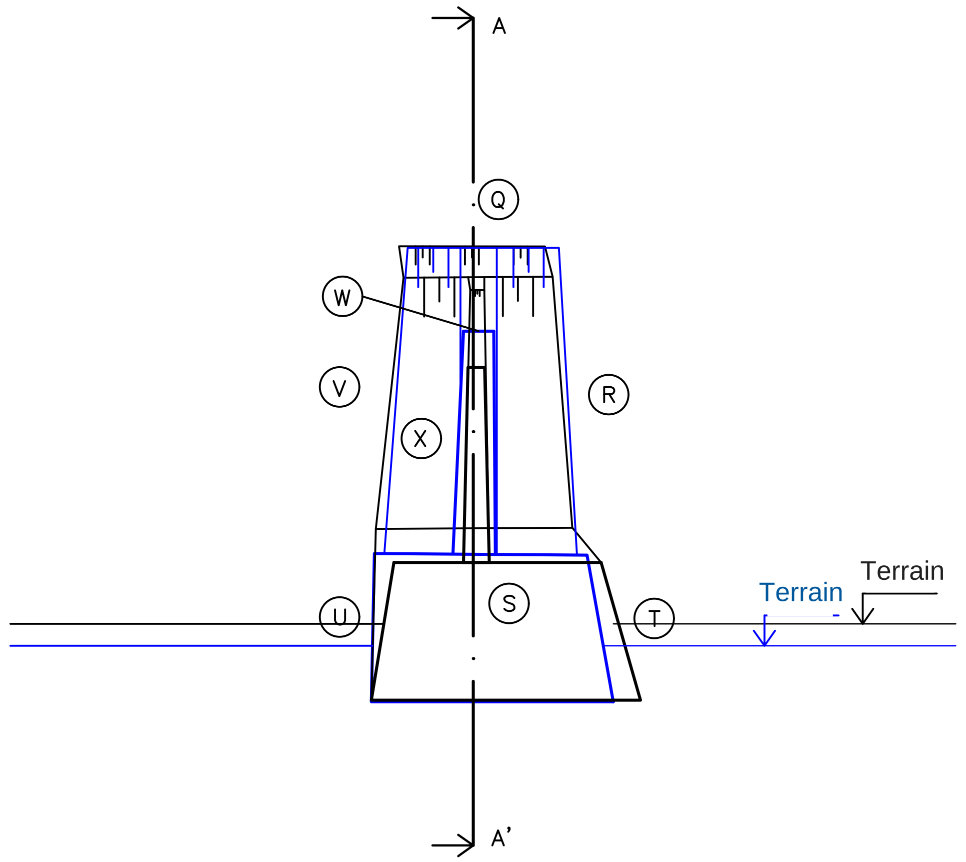

FLOOR PLAN

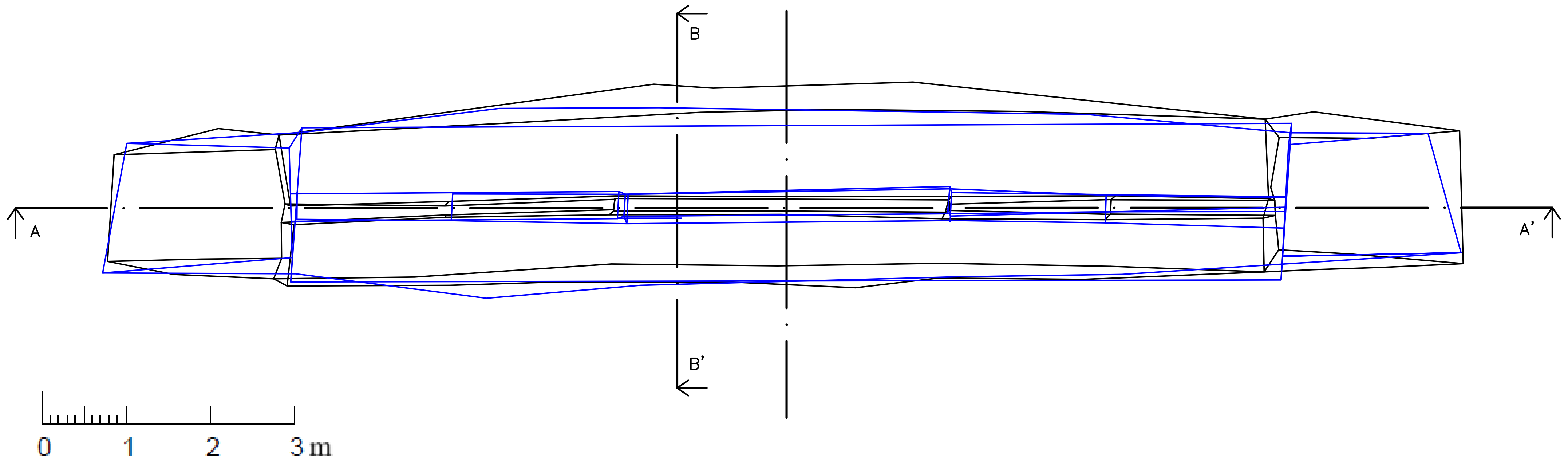

Mark	Total station	iPhone 14 Pro	Deviation	Deviation %
Ⓐ	3 000 mm	3 000 mm	0 mm	0.0 %
Ⓑ	1 599 mm	1 690 mm	−91 mm	5.7 %
Ⓒ	2 970 mm	2 998 mm	−28 mm	0.9 %
Ⓓ	561 mm	503 mm	58 mm	10.3 %
Ⓔ	2 948 mm	3 010 mm	−62 mm	2.1 %
Ⓕ	605 mm	510 mm	95 mm	15.7 %
Ⓖ	3 040 mm	2 953 mm	87 mm	2.9 %
Ⓗ	1 483 mm	1 706 mm	−223 mm	15.0 %
Ⓘ	1 904 mm	1 674 mm	230 mm	12.1%
Ⓙ	3 012 mm	2 960 mm	52 mm	1.7 %
Ⓚ	1 463 mm	1 318 mm	145 mm	9.9 %
Ⓛ	2 940 mm	3 020 mm	−80 mm	2.7 %
Ⓜ	1 486 mm	1 409 mm	77 mm	5.2 %
Ⓝ	3 041 mm	2 911 mm	130 mm	4.3 %
Ⓞ	2 033 mm	1 807 mm	226 mm	11.1%
Ⓟ	3 000 mm	3 000 mm	0 mm	0.0 %
Ⓠ	1 000 mm	965 mm	35 mm	3.5 %
Ⓡ	2 034 mm	2 122 mm	−88 mm	4.3 %
Ⓢ	1 407 mm	1 370 mm	37 mm	2.6 %
Ⓣ	985 mm	927 mm	57 mm	5.8 %
Ⓤ	980 mm	922 mm	58 mm	5.9 %
Ⓥ	2 028 mm	2 037 mm	−9 mm	0.4 %
Ⓦ	200 mm	110 mm	90 mm	45.0 %
Ⓧ	1 470 mm	1 290 mm	180 mm	12.2 %
			Mean	7.0 %

Total station

iPhone 14 Pro
