# Supplementary material for: Accuracy Assessment of iPhone LiDAR for Mapping Streambeds and Small Water Structures in Forested Terrain
Source: Sensors (Basel). 2025 Oct 4;25(19):6141. doi: 10.3390/s25196141 (PMC12526706; doi:10.3390/s25196141)
Supplement: Supplementary file 1 [file sensors-25-06141-s001.zip › S2_structure_2.pdf]

SECTION A - A´

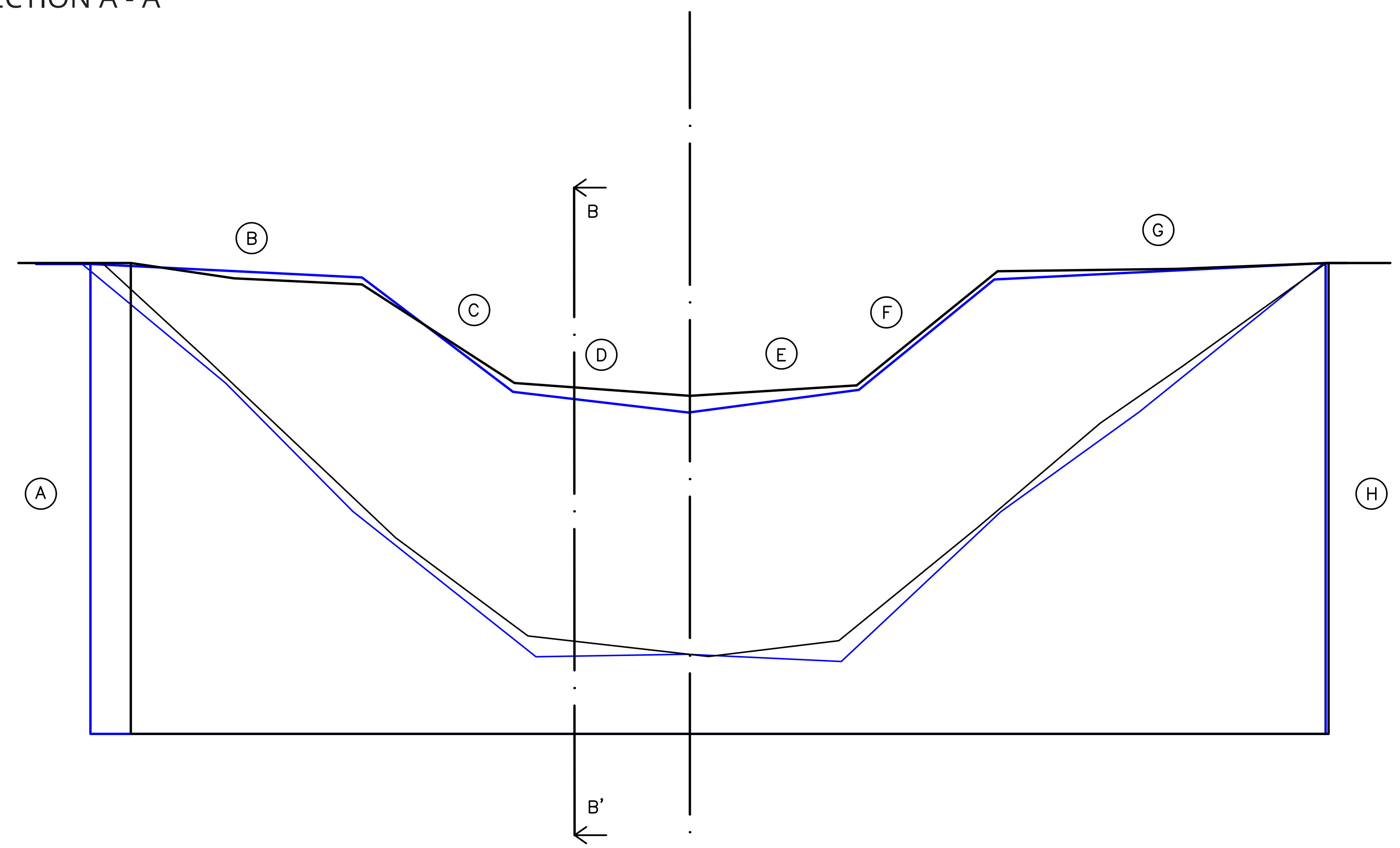

SECTION B - B´

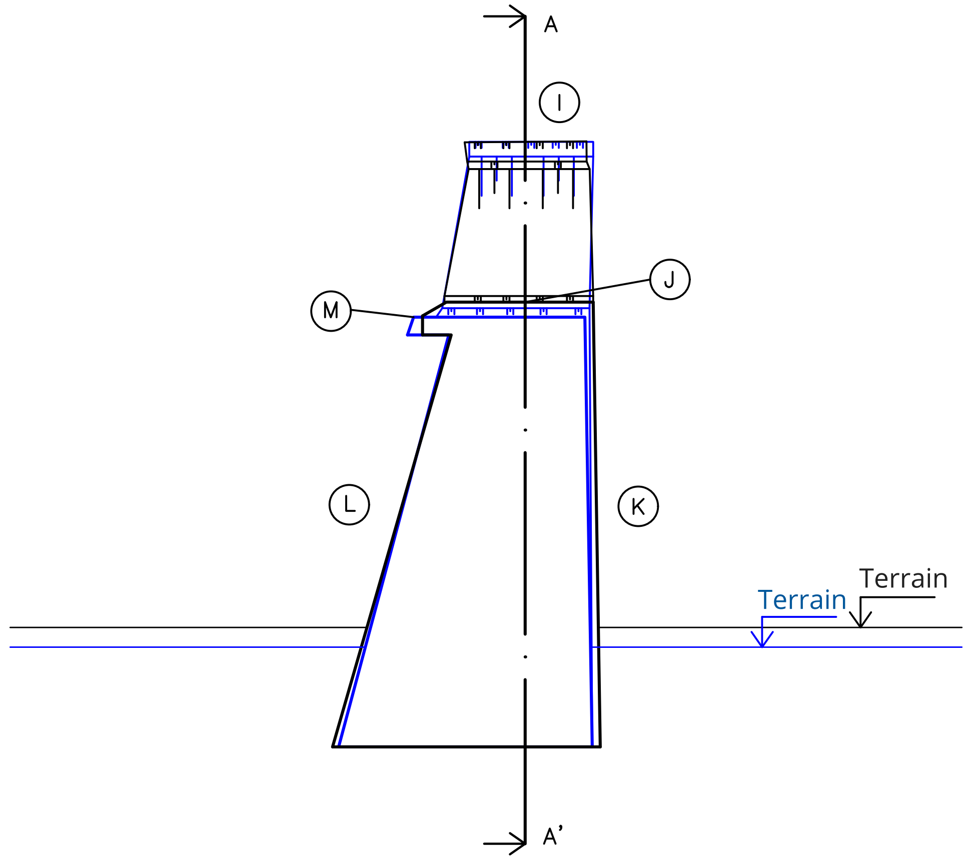

Mark	Total station	iPhone 14 Pro	Deviation	Deviation %
Ⓐ	3 992 mm	4 000 mm	−8 mm	3.7 %
Ⓑ	2 309 mm	1 973 mm	336 mm	15.6 %
Ⓒ	1 609 mm	1 542 mm	67 mm	4.2 %
Ⓓ	1 500 mm	1 496 mm	4 mm	0.3 %
Ⓔ	1 460 mm	1 420 mm	40 mm	2.7 %
Ⓕ	1 485 mm	1 544 mm	−59 mm	4.0 %
Ⓖ	3 085 mm	2 813 mm	272 mm	8.8 %
Ⓗ	4 000 mm	4 000 mm	0 mm	0.0 %
Ⓘ	820 mm	805 mm	15 mm	1.8 %
Ⓙ	1 178 mm	1 133 mm	45 mm	3.8 %
Ⓚ	2 841 mm	2 940 mm	−99 mm	3.5 %
Ⓛ	2 819 mm	2 834 mm	−15 mm	0.5 %
Ⓜ	123 mm	269 mm	−144 mm	116.1 %
			Mean	12.4 %

FLOOR PLAN

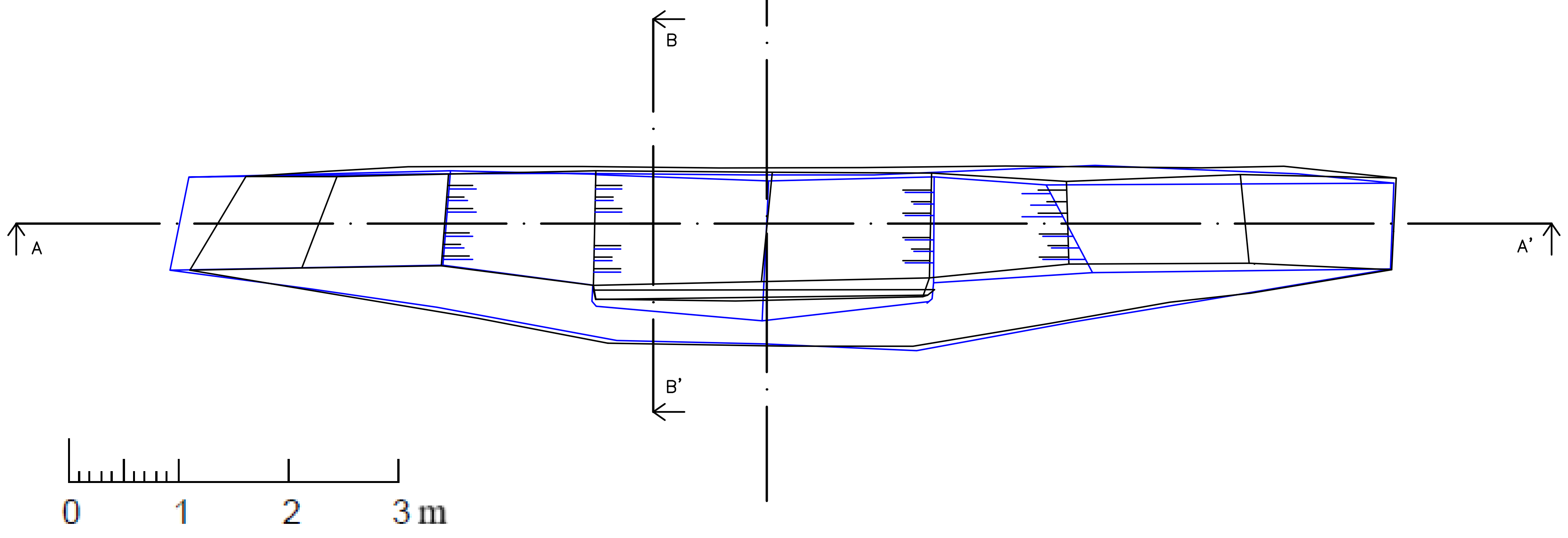

Total station

iPhone 14 Pro
