# Supplementary material for: Accuracy Assessment of iPhone LiDAR for Mapping Streambeds and Small Water Structures in Forested Terrain
Source: Sensors (Basel). 2025 Oct 4;25(19):6141. doi: 10.3390/s25196141 (PMC12526706; doi:10.3390/s25196141)
Supplement: Supplementary file 1 [file sensors-25-06141-s001.zip › S3_structure_3.pdf]

SECTION A - A´

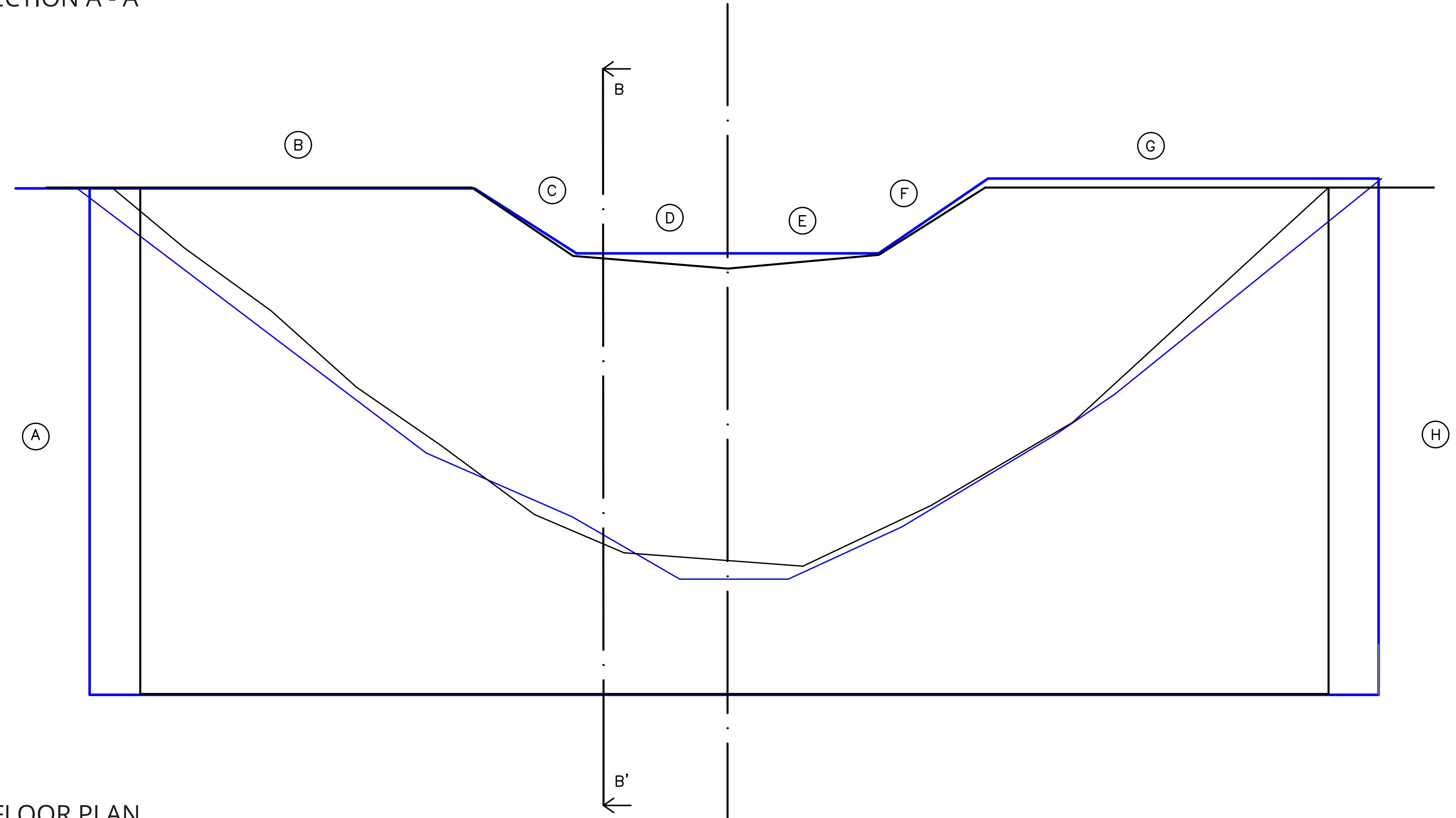

SECTION B - B´

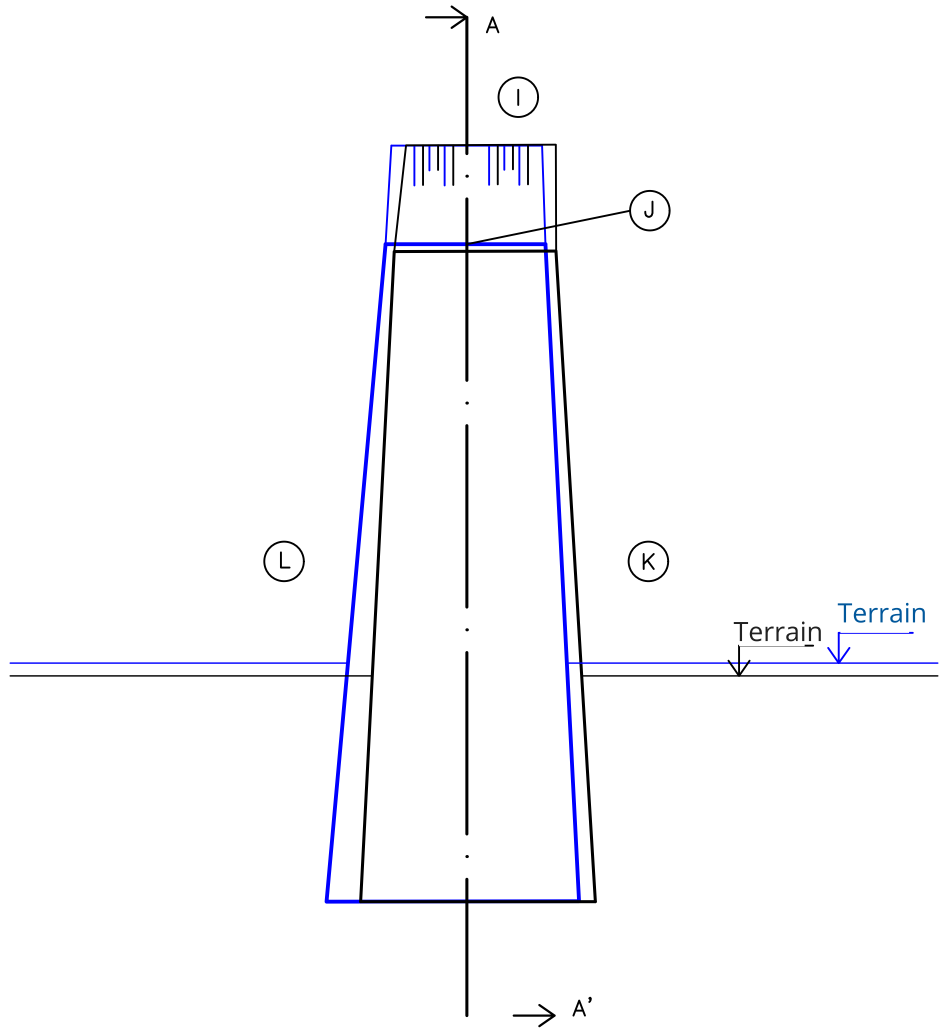

Mark	Total station	iPhone 14 Pro	Deviation	Deviation %
Ⓐ	5 000 mm	5 000 mm	0 mm	0.0 %
Ⓑ	3 799 mm	3 390 mm	409 mm	10.8 %
Ⓒ	1 007 mm	1 050 mm	−43 mm	4.3 %
Ⓓ	1 492 mm	1 485 mm	7 mm	0.5 %
Ⓔ	1 492 mm	1 535 mm	−43 mm	2.9 %
Ⓕ	1 080 mm	1 000 mm	80 mm	7.4 %
Ⓖ	3 857 mm	3 273 mm	584 mm	15.1 %
Ⓗ	5 097 mm	5 000 mm	97 mm	1.9 %
Ⓘ	990 mm	990 mm	0 mm	0.0 %
Ⓙ	1 056 mm	1 170 mm	−114 mm	10.8 %
Ⓚ	4 475 mm	4 308 mm	167 mm	3.7 %
Ⓛ	4 494 mm	4 306 mm	188 mm	4.2 %
			Mean	5.0 %

FLOOR PLAN

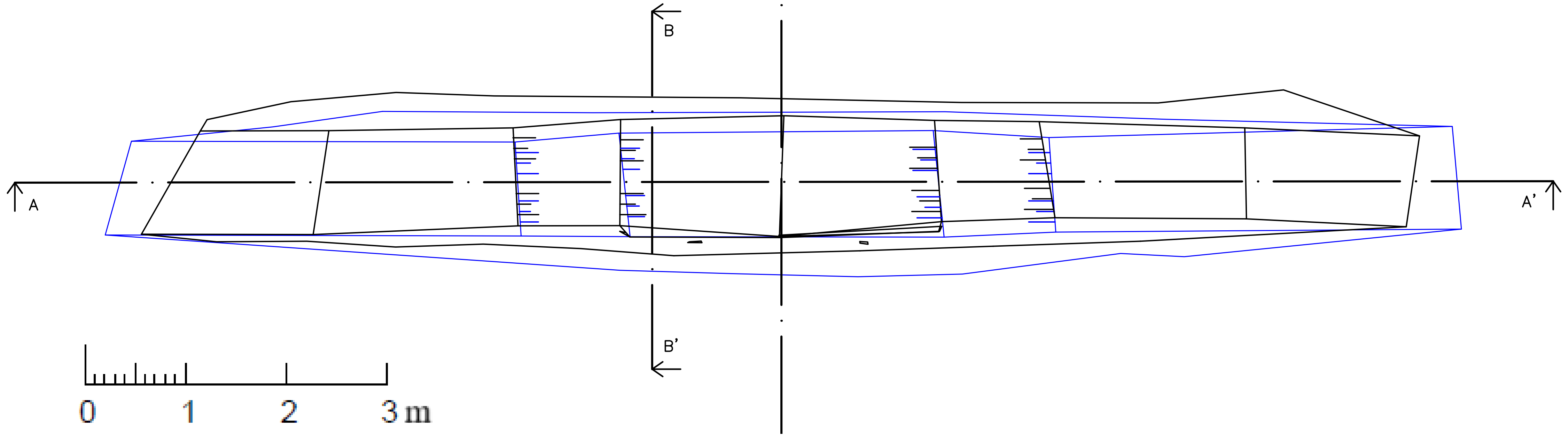

Total station

iPhone 14 Pro
